# Supplementary material for: Novel Driver Strength Index highlights important cancer genes in TCGA PanCanAtlas patients
Source: PeerJ. 2022 Aug 11;10:e13860. doi: 10.7717/peerj.13860 (PMC9375969; doi:10.7717/peerj.13860)
Supplement: Supplemental Information 2 [file peerj-10-13860-s002.zip › COHORTS/genes plots/cohorts/2021_8_16_14_9_distribution_cohorts_females_BLCA.pdf]

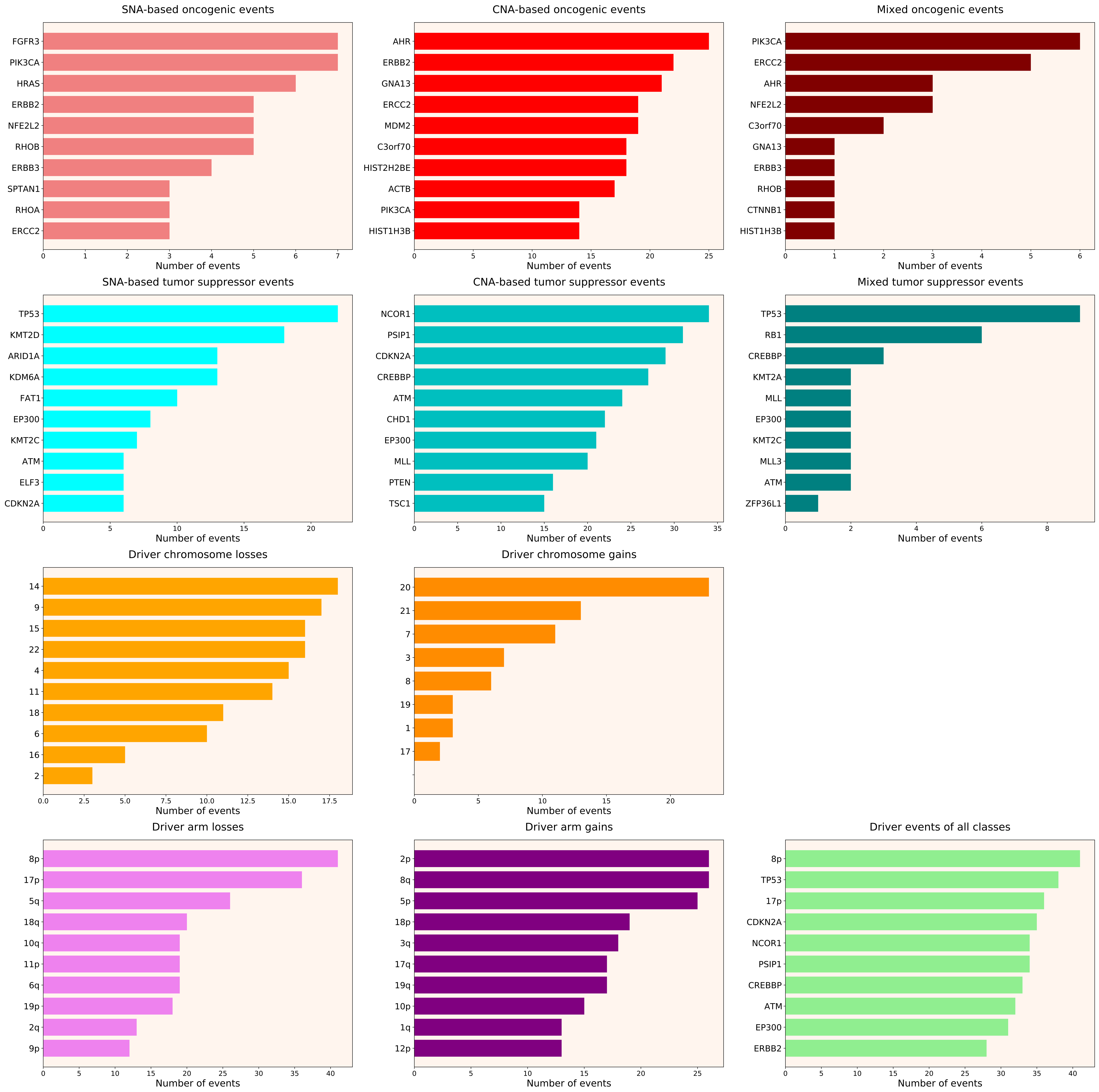
SNA-based oncogenic events
FGFR3
PIK3CA
HRAS
ERBB2
NFE2L2
RHOB
ERBB3
SPTAN1
RHOA
ERCC2
Number of events
CNA-based oncogenic events
AHR
ERBB2
GNA13
ERCC2
MDM2
C3orf70
HIST2H2BE
ACTB
PIK3CA
HIST1H3B
Number of events
Mixed oncogenic events
PIK3CA
ERCC2
AHR
NFE2L2
C3orf70
GNA13
ERBB3
RHOB
CTNNB1
HIST1H3B
Number of events
SNA-based tumor suppressor events
TP53
KMT2D
ARID1A
KDM6A
FAT1
EP300
KMT2C
ATM
ELF3
CDKN2A
Number of events
CNA-based tumor suppressor events
NCOR1
PSIP1
CDKN2A
CREBBP
ATM
CHD1
EP300
MLL
PTEN
TSC1
Number of events
Mixed tumor suppressor events
TP53
RB1
CREBBP
KMT2A
MLL
EP300
KMT2C
MLL3
ATM
ZFP36L1
Number of events
Driver chromosome losses
14
9
15
22
4
11
18
6
16
2
Number of events
Driver chromosome gains
20
21
7
3
8
19
1
17
Number of events
Driver arm losses
8p
17p
5q
18q
10q
11p
6q
19p
2q
9p
Number of events
Driver arm gains
2p
8q
5p
18p
3q
17q
19q
10p
1q
12p
Number of events
Driver events of all classes
8p
TP53
17p
CDKN2A
NCOR1
PSIP1
CREBBP
ATM
EP300
ERBB2
Number of events
